# A Plea for More Laboratory Work

**Published:** 1906-08

**Authors:** E. C. Thrash

**Affiliations:** Atlanta, Ga.; Professor of Pathology and Bacteriology Atlanta School of Medicine


### A PLEA FOR MORE LABORATORY WORK.

By E. C. THRASH, M.D., Atlanta, Ga.
Professor of Pathology and Bacteriology Atlanta School of Medicine.

Laboratory work has always been surrounded with entirely too much glamor by the average practitioner, and there is too great a tendency to associate it with a well-equipped college or hospital laboratory, feeling that good results can only be had at these places. They shun the work as something incomprehensible or too difficult, and giving too little results in return for the outlay of labor and equipments that are required. The sooner this is eliminated from the physician's mind, and the sooner he will spend a little money to equip a corner in his office to do his own work the quicker he will become a more painstaking, and a more thorough diagnostician, and be made more satisfied with his own work. Some of the most profound pathological research that has ever been made was accomplished by men with most meager and unpretentious equipments in the most obscure places. The object of this paper, however, is not to advise men in general practice to make research, but to show how satisfactorily the practical part of this work can be done, and how it will help him in difficult and doubtful cases.

What the writer intends to outline is not an untried theory, but what he has put into practice for many years. Time will not permit the details of technique being given here, and besides such a procedure would be entirely out of place in a paper of this length. It is not expensive to equip one's office for work of this kind, and the thorough working out of even one case may repay him for the entire investment. An outlay of $150 will get an outfit, with which as good results can be had as is needed for all practical purposes, and even this price can be shaved if necessary. There are a great many complicated devices for which simple substitutes can be made to answer well. To illustrate, a tin box, a thermometer, and an alcohol lamp can be used for a drying oven; a good razor for a microtome; a small closet, and a lamp for an incubator, etc. What a man wants is results, and if he gets them with an improvised device, he is as well off as if he had gotten them at the best regulated laboratory in the world. Physicians are too prone to say that they do not have time to do this class of work. It is not so. The great majority of them do have time. It is a diversion, and lends interest to one's labors. One feels much better, knowing he is doing scientific work, than to feel that he is merely drifting, and indulging in slipshod methods.

In the following diseases the microscope is almost indispensable: Tuberculosis, typhoid fever, malaria, diphtheria, nephritis, the primary anemias, gonorrhoea, tumors and intestinal parasites. It is valuable in many other diseases, but these embrace the everyday work of general medicine. Think of the importance of this array of diseases. Look over your list of cases for the past year, five years, or since you have been practicing and note what a large percentage of all organic diseases are found in this list, and then think of what aid a microscope would be in each. This is an old, old story, but my object is to emphasize what we already know. We frequently know a thing all our lives, and never get thoroughly awakened to its importance. Unless one wishes he need not go any deeper into laboratory work than just the study of the diseases above mentioned. In these he will get the most of practical clinical microscopy. On the other hand, until he does accomplish this, he lacks to the degree in which he is deficient, being a thorough diagnostician. These things are entirely too simple to go through life stumbling over them. To illustrate—examination for tubercle bacilli is as easy as making a test for sugar in urine. Examination for the gonococcus is almost as simple as getting and obtaining reaction urine. In fact, the laboratory technique for the study of all these diseases is so uncomplicated that one will be surprised at the facility with which he can do it, if just a little time will be spent in preparation.

My plea, gentlemen, is for thoroughness in diagnosis. We may go on treating cases with indifference as to the condition, telling people they have "bronchial trouble," "stomach trouble," "kidney trouble," "biliousness," and other vague afflictions, and succeed to the entire satisfaction of our patients, but what we need is to be always dissatisfied with ourselves. The man who makes an early diagnosis of a disease, say tuberculosis, and starts his patient in the direction of a cure, is doing much better work than he who treats the same kind of case and calls it "bronchial trouble," telling the patient that if he is not extremely careful it may run into consumption, never making a clear diagnosis until the poor sufferer is in the throes of death.

Another illustration, given a febrile condition. Good work is to get busy at once, and not only study the case at the bedside, but make such laboratory tests as are necessary to assist in arriving at a definite conclusion at as early a date as possible, making it plain to the family that the case is being studied, and when information is given let it be that which can be stood by to the end. Inferior work is telling a man in a similar condition, first, that he is bilious, and later, when he continues ill, that he has contracted some malaria, then still later that he has typho-malarial fever, and after the case does not clear up within three or four weeks, that the case has run into the "regular old typhoid fever." In the meantime allowing enough germs of typhoid fever to be scattered around the premises to infect not only all the rest of the family, but all the neighbors, with plenty to spare to the casual visitor.

Another illustration of good work: Given a case that has gone the rounds, and been treated for anemia, indigestion, malnutrition, dirt-eating, and what not. Examine the feces for uncinaria, which will likely be found, cure him and make the family happy. Inferior work would be to examine the tongue, listen to the heart, feel the pulse, and write a prescription. Every doctor knows of men who are doing each of these classes of work, although it is the duty of all men who practice medicine to give the world the best that is in them.

### DISCUSSION OF DR. THRASH'S PAPER.

*Dr. C. A. Smith:* I have listened with a great deal of pleasure to the paper by Dr. Thrash. It is true that a doctor can fit up a small laboratory in one corner of his office at an extremely small expense. Of course, he can do only a limited amount of work, but where he will take the trouble to keep up with the simple examinations for the more common diseases, he will be more than repaid in the diagnosis of his cases. Most of the apparatus can be improvised at very little expense, the most expensive part of the equipment being the microscope, and in purchasing this instrument he should always be sure to get the best.

While it is a comparatively simple matter to make many of these examinations, yet my observation has been that many men who equip themselves for this work do not find time to keep it up.

